# Low birth weight associates with glomerular area in young male IgA nephropathy patients

**DOI:** 10.1186/s12882-018-1070-7

**Published:** 2018-10-22

**Authors:** Paschal Ruggajo, Sabine Leh, Einar Svarstad, Hans-Peter Marti, Bjørn Egil Vikse

**Affiliations:** 1 Department of Internal Medicine, Muhimbili University of Health and Allied Sciences (MUHAS), P.O.Box 65001, Dar es Salaam, Tanzania; 2 Department of Clinical Medicine, University of Bergen, Bergen, Norway; 3 Department of Medicine, Haukeland University Hospital, Bergen, Norway; 4 Department of Pathology, Haukeland University Hospital, Bergen, Norway; 5 Department of Medicine, Haugesund Hospital, Haugesund, Norway

## Abstract

**Background:**

In a recent study we demonstrated that low birth weight (LBW) was associated with increased risk of progressive IgA nephropathy (IgAN). In the present study we investigate whether this could be explained by differences in glomerular morphological parameters.

**Methods:**

The Medical Birth Registry of Norway has registered all births since 1967 and the Norwegian Kidney Biopsy Registry has registered all kidney biopsies since 1988. Patients diagnosed with IgAN, registered birth weight and estimated glomerular filtration rate above 60 ml/min/1.73m^2^ at time of diagnosis were eligible for inclusion. Patients were included in a case-control manner based on whether or not they had LBW or were small for gestational age (SGA). Glomerular area, volume and density were measured using high resolution digital images and differences were compared between groups.

**Results:**

We included 51 IgAN patients with a mean age of 23.6 years, 47.1% male. Compared to IgAN patients without LBW or SGA, IgAN patients with LBW and/or SGA had larger glomerular area (16,235 ± 3744 vs 14,036 ± 3502 μm^2^, *p*-value 0.04). This was significant for total cohort and male but not female. On separate analysis by gender, glomerular area was significantly larger only in males (17,636 ± 3285 vs 13,346 ± 2835 μm^2^, *p*-value 0.004). Glomerular density was not different between groups. In adjusted linear regression analysis, glomerular area was negatively associated with birth weight.

**Conclusion:**

Among young adult IgAN patients, low birth weight is associated with having larger glomerular area, especially in males. Larger glomeruli may be a sign of congenital nephron deficit that may explain the increased risk of progressive IgAN.

**Electronic supplementary material:**

The online version of this article (10.1186/s12882-018-1070-7) contains supplementary material, which is available to authorized users.

## Background

Brenner and co-workers suggested that adverse intrauterine environment, for example due to placental insufficiency or maternal undernutrition, was associated with impaired nephron development and increased risk of hypertension and progressive kidney disease in adult life [1]. Birthweight related parameters such as low birth weight (LBW) and small for gestational age (SGA) are strong surrogate markers for adverse intrauterine environment [2, 3]. LBW has been associated with increased risk of hypertension [4], albuminuria [5, 6] and progressive chronic kidney disease [7, 8]. Fewer studies have investigated effects of SGA, but in a previous study from Norway we showed that SGA was a stronger risk marker than LBW for development of ESRD in adult age [9].

IgA nephropathy (IgAN) is the most frequent primary idiopathic glomerulonephritis worldwide [10–12] and has a variable clinical course [13–15]. In a previous study we showed that LBW and SGA were risk factors for progression to ESRD in IgAN patients [16].

Several previous autopsy-based stereological and histomorphometric studies have reported strong correlations between LBW and reduced glomerular numbers as well as increased glomerular volume [17–19]. A previous study has also shown that IgAN patients born SGA had increased risk for glomerulosclerosis and arterial hypertension [20]. To our knowledge, the association between birth weight and glomerular histomorphometric changes among IgAN patients has not been investigated before.

In the present study we aimed to investigate how LBW and SGA in IgAN patients associate with specific glomerular morphological changes compared to IgAN without LBW and SGA. We hypothesized that LBW and SGA would be associated with fewer and larger glomeruli in line with the Brenner hypothesis but that the effects of LBW and SGA could be different. In our previous study cited above, associations were stronger in males, so gender differences were also investigated in the present study.

## Methods

### Description of registries

Since 1967, the Medical Birth Registry of Norway has registered extensive medical data on all births in Norway (total population of 5.1 million) [21]. The Norwegian Kidney Biopsy Registry has registered clinical and histopathological data (including diagnosis) for all patients who have had a kidney biopsy performed in Norway since 1988. Since 1980, the Norwegian Renal Registry has registered data on all patients in Norway who developed ESRD (defined as starting maintenance dialysis treatment or undergoing renal transplantation). The data from all registries were available until December 2013 and data were linked using the unique 11-digit national identification number.

The regional ethics committee of Western Norway gave clearance for the study (2013/553), participants were asked for consent by mail.

### Variables from the medical birth registry and Norwegian kidney biopsy registry

LBW was defined as birth weight less than the 10th percentile for gender (2930 g for male, 2690 g for female) in the study population of our previous study [16]. From 1967 through 1998, gestational age was based on the last menstrual period and from 1999 onwards on routine ultrasonographic examination in gestational weeks 17 through 20. Based on national data on birth weight, gestational week, gender and plurality, a z-score denoting numbers of standard deviations from mean of birth weight for each week of gestational age was calculated for all cases and controls based on data from the Medical Birth Registry [22, 23]. Small for gestational age (SGA) was defined as birth weight less than the 10th percentile for gestational week in the study population of our previous study [16] (defined by z-score less than − 1.2900 for male and − 1.5280 for female gender). Preterm birth was defined as a gestational age less than 37 weeks [22, 23].

Maternal pregestational disease was defined as maternal rheumatic disease, renal disease, diabetes mellitus or hypertension diagnosed before pregnancy and reported at birth to the Medical Birth Registry [24]. Maternal pre-eclampsia was defined based on the 1972 recommendations of the American College of Obstetricians and Gynecologists, i.e. hypertension and proteinuria after week 20 of gestation [25].

The following clinical variables at the time of kidney biopsy were used in the present study; estimated glomerular filtration rate (eGFR), calculated using the IDMS-traceable CKD-EPI equation [26]. All patients were assumed to be of Caucasian race. For patients who had a kidney biopsy performed before year 2005, their serum creatinine levels were reduced by 5% to standardize them to IDMS-traceable levels [27]. To avoid spurious eGFR distribution in patients with very low values of serum creatinine, all eGFR values exceeding 150 ml/min/1.73m^2^ were set to 150 ml/min/1.73m^2^. Proteinuria (grams/24 h), systolic and diastolic blood pressure measurements (mmHg) at time of biopsy were all used as continuous variables.

### Selection of cases and controls

We selected cases and controls from patients registered in the described registries (Additional file 1: Figure S1). Eligible patients had registered birth weight, eGFR > 60 ml/min/1.73m^2^ at time of biopsy and did not develop ESRD during follow-up (as registered in the Norwegian Renal Registry). The last two criteria were chosen to ensure that during selection, our study population comprised of patients with lowest risk of progressive IgAN population at the time of biopsy (in addition, only 1 eligible patient had developed ESRD). Power calculations were based on extrapolations from two human studies which had published measurements of glomerular size in relation to birth weight [17, 18]. From these eligible patients, we selected 5 groups of patients:(1) 20 patients randomly selected out of the 230 eligible patients registered in the Kidney Biopsy Registry with a diagnosis of IgAN and being neither LBW nor SGA, (2) all 13 patients with LBW but not SGA, (3) all 15 patients with SGA but not LBW and (4) all 14 patients with both LBW and SGA. As some of the selected patients did not have available kidney biopsy tissue, we were able to include 18, 11, 10 and 12 case patients in these respective groups. In addition, (5) we selected 20 patients who were neither LBW nor SGA, had normal findings on kidney biopsy (indicated for proteinuria or haematuria) and had eGFR > 60 ml/min/1.73m^2^ as normal controls; this control group was age-and-sex matched with the group of IgAN cases without LBW or SGA; 19 patients in this control group had available tissue and were included.

### Histopathological and histomorphometric variables

Representative periodic acid Schiff stained 3 μm thick sections from paraffin embedded formalin fixed tissue were used. Morphological evaluation was done on digital slides scanned at 40x resulting in a resolution of 0.25 μm per pixel (Aperio ScanScope™ XT System, 1360 Park Center Drive Vista, CA 92081, USA). The digital images were viewed in Imagescope (version 12, Leica/Aperio). We recorded the total number of glomeruli, the number of glomeruli with mesangial hypercellularity, endocapillary hypercellularity, global sclerosis, segmental sclerosis and tubular atrophy as defined by the Oxford classification for IgAN and a MEST score was calculated [28]. The annotation pen tool was used to measure glomerular tuft areas and cortical area of each profile. We determined the glomerular density as a ratio of number of glomeruli per cortical area, and this was calculated for all glomeruli (reported as total glomerular density), for sclerosed glomeruli and for non-sclerosed glomeruli. We used total glomerular density during analysis. The glomerular tuft area was defined as an area bound by outer capillary loops of the glomerular tuft and the mean glomerular area was obtained by averaging the area of all eligible glomeruli in the profile. Glomerular diameter (d) was calculated from the formula d = square root of (4A/π), where A = glomerular area and π = 3.142, assuming the glomerular tuft to be circular in shape.

For estimating glomerular volume we used the Weibel-Gomez formula expressed as GV = (GA)^3/2^x β/d; where GV = glomerular volume, GA = glomerular tuft area, β = 1.38 (assuming the glomerulus is spherical in shape) and d is a size distribution coefficient that is used to adjust for the variation in glomerular size; we used d = 1.01 as adopted from other studies [29–31]. The microscopic and morphometric evaluations for all kidney biopsies in this study were performed by the first author after thorough training by an experienced nephropathologist (second author) who also verified all measurements. These evaluations were blinded for information on LBW / SGA.

### Statistical analysis

Birth weight related parameters were used as exposure variables whereas histopathological and histomorphometric parameters were used as outcome variables. In the primary analyses, outcome variables were compared between the four sub-groups with IgAN described above; in further analyses all groups with LBW and/or SGA were combined. Also, IgAN patients without LBW or SGA were compared to control patients. Continuous variables were tested for normal distribution (confirmed by P-P plots for all glomerular size parameters) and compared using student t-tests while categorical variables were compared using Chi-square tests. Linear regression statistics were used to determine the association between glomerular area and birthweight-related variables and clinical variables. In the primary analysis (model 1) we adjusted for diagnostic group (IgA nephropathy vs control) as the study sample consisted of these two separate groups. In the secondary analysis (model 2) we further adjusted for gender and age at biopsy. If not otherwise stated, values are reported as mean ± standard deviation; *P* < 0.05 was considered statistically significant, and all tests were 2 tailed. Analyses were performed using the SPSS version 21 (SPSS, Chicago, IL).

## Results

A total of 51 patients with IgAN were included in the present study, of these 24 (47.1%) were males. Mean age at biopsy was 23.6 years and mean eGFR was 95.3 ml/min/1.73m^2^. At the time of kidney biopsy, patients without LBW or SGA had comparable clinical characteristics to patients with LBW and/or SGA (Table 1). As expected, birth weight characteristics varied between groups.

**Table 1:** Cohort characteristics among Cases and Controls in Norway, 1988–2013

	IgAN controls	IgAN cases			IgAN cases combined
	Neither LBW nor SGA	LBW but not SGA	SGA but not LBW	Both LBW and SGA	LBW and/or SGA
N	18	11	10	12	33
N (%) male	8 (44.4)	5 (45.5)	5 (50.0)	6 (50.0)	16 (48.5)
Age at Biopsy (years)	22.8 ± 9.0	21.1 ± 8.6	22.3 ± 7.1	28.3 ± 8.9	24.1 ± 8.7
Weight at Biopsy (Kg)	76.8 ± 22.6	56.8 ± 27.9	68.3 ± 18.6	75.2 ± 14.3	66.1 ± 21.8
Systolic BP (mmHg)	124.4 ± 22.9	118.8 ± 16.2	131.3 ± 9.8	126.1 ± 17.5	124.8 ± 15.6
Diastolic BP (mmHg)	76.6 ± 13.2	72.6 ± 14.9	77.8 ± 10.1	72.7 ± 11.1	74.0 ± 12.2
eGFR (ml/min/1.73m^2^)	94.8.0 ± 27.3	99.4 ± 32.9	95.5 ± 23.5	92.1 ± 18.6	95.5 ± 25.0
Urinary Protein (g/d)	1.8 ± 1.7	1.7 ± 1.5	1.0 ± 1.3	2.4 ± 2.6	1.7 ± 1.9
Birth Weight (kg)	3.6 ± 0.53	1.9 ± 0.75^b^	2.9 ± 0.17^a^	2.4 ± 0.42^b^	2.4 ± 0.65^b^
Gestational age (week)	40.3 ± 1.1	32.6 ± 4.5^b^	40.40 ± 0.70	38.7 ± 1.2^b^	37.2 ± 4.3^b^
Birthweight for Gestational Age (Z-score)	−0.18 ± 1.04	− 0.28 ± 0.73	−1.56 ± 0.24^b^	−2.19 ± 0.54^b^	− 1.36 ± 0.98^b^
Maternal Pre-eclampsia	0 (0.0)	0 (0.0)	2 (20.0)	2 (16.7)	4 (12.1)
Maternal pregestational disease^b^	0 (0.0)	0 (0.0)	0 (0.0)	1 (8.3)	1 (3.0)

^a^pvalue< 0.01,^b^p-value< 0.001 as compared to IgAN controls

^b^Maternal pregestational disease was defined as maternal rheumatic disease, renal disease, diabetes mellitus or hypertension

### Birth weight related variables and glomerular morphological changes

As shown in Table 2, glomerular measurements and other morphological variables were similar between groups with LBW and/or SGA (explored with t-tests between groups), these groups were therefore combined in the main analysis. Glomerular morphology, as investigated with markers of glomerular damage and the Oxford classification, were similar between groups. In glomerular histomorphometric analysis, compared to IgAN patients without LBW or SGA, IgAN patients with LBW and/or SGA had larger glomerular area (16,235 ± 3744 vs 14,036 ± 3502 μm^2^, *p*-value 0.004). Further analyses stratified by gender showed that glomerular area was significantly larger only in males (17,636 ± 3285 vs 13,346 ± 2835 μm^2^, p-value 0.004) but not in females (14,588 ± 4018 vs 14,918 ± 3758 μm^2^, p-value 0.8). Glomerular density was not different between groups. In a sensitivity analysis, we tested whether including only patients with 9 glomeruli or more [32] would change the results for glomerular area, in these analyses the differences in glomerular area were comparable, but due to a lower N it did not reach statistical significance. In an analysis in which the two groups with LBW (excluding the group with only SGA) were compared to the group without LBW and SGA, glomerular area was statistically significantly different (14,036 vs 16,383 μm^2^, *p*-value 0.04).

**Table 2:** Glomerular histopathological and histomorphometric variables stratified by LBW and/or SGA, Norway (1988–2013)

	IgAN Controls Without	IgAN Cases			IgAN cases combined
	LBW or SGA	LBW but not SGA	SGA but not LBW	Both LBW and SGA	LBW and/or SGA
N	18	11	10	12	33
N (%) with M-score of 1	10 (55.6)	8 (72.7)	5 (50.0)	4 (33.3)	17 (51.5)
N (%) with E-score of 1	3 (16.7)	2 (18.2)	0 (0.0)	1 (8.3)	3 (9.1)
N (%) with S-score of 1	1 (5.6)	3 (27.3)	2 (20.0)	1 (8.3)	6 (18.2)
N (%) with T-score of 1	0 (0.0)	0 (0.0)	0 (0.0)	0 (0.0)	0 (0.0)
% of glomeruli with mesangial hypercellularity	49.6 ± 27.4	49.1 ± 35.6	39.8 ± 21.7	35.0 ± 29.7	41.2 ± 29.4
% of glomeruli with endocapillary hypercellularity	0.93 ± 2.7	7.3 ± 20.1	0.00 ± 0.00	2.0 ± 6.8	3.2 ± 12.3
% of glomeruli with global sclerosis	3.3 ± 6.9	12.8 ± 29.6	3.3 ± 6.2	2.5 ± 5.9	5.9 ± 17.8
% of glomeruli with segmental sclerosis	0.69 ± 2.9	2.5 ± 5.2	1.6 ± 3.3	0.25 ± 0.85	1.5 ± 3.6
Histomorphometric variables					
Number of glomeruli (N)	14.1 ± 7.8	16.0 ± 7.5	16.2 ± 6.4	14.7 ± 11.4	15.6 ± 8.6
Glomerular tuft volume (μm^3^x10^6^) ^c^	2.37 ± 0.82	2.90 ± 0.97	2.81 ± 1.13	2.93 ± 0.89	2.89 ± 0.96
Glomerular tuft area (μm^2^)	14,036 ± 3502	16,311 ± 3805	15,899 ± 4335	16,447 ± 3478	16,235 ± 3744^a^
Glomerular tuft diameter (μm) ^c^	132.7.4 ± 16.8	143.1. ± 17.5	141.1 ± 19.5	143.9 ± 15.9	142.8 ± 17.0^a^
Glomerular density (N per 10^6^ μm^2^)	2.95 ± 0.85	3.54 ± 1.30	3.36 ± 0.93	2.66 ± 1.47	3.17 ± 1.30

^a^p-value< 0.01,^b^p-value< 0.001 as compared to IgAN controls

^c^ Glomerular tuft volume and glomerular tuft diameter was calculated based on the measured glomerular tuft area (as described in detail in the methods section)

### Comparison between patients with normal biopsy and IgAN

To investigate whether development of IgAN altered glomerular area, we compared results for patients with IgAN who did not have LBW or SGA (*N* = 18) to a matched group of control patients with normal biopsies (*N* = 19). As shown in Table 3, clinical characteristics were comparable between these two groups except for proteinuria which was significantly higher among the IgAN controls (1.8 ± 1.7 vs 0.32 ± 0.28 g/d, *p*-value 0.002). There were no significant differences between birth weight-related and glomerular histomorphometric characteristics observed between the two groups but there was a non-significant trend towards larger glomeruli in IgAN patients.

**Table 3:** Comparison of clinical, birth-related and glomerular histomorphometric characteristics between normal controls and IgAN controls

	Normal controls	IgAN controls	
	Not LBW or SGA	Not LBW or SGA	p-value
N	19	18	
N (%) Male	10 (52.6)	8 (44.4)	1.0
Age at Biopsy (years)	21.8 ± 8.6	22.8 ± 9.0	0.7
Systolic BP (mmHg)	114.7 ± 15.6	124.4 ± 22.9	0.1
Diastolic BP (mmHg)	71.3 ± 13.8	76.6 ± 13.2	0.2
eGFR (ml/min/1.73m^2^)	92.5 ± 28.4	94.8 ± 27.3	0.8
Urinary protein (g/d)	0.32 ± 0.28	1.8 ± 1.7^a^	0.002
Birth Weight (kg)	3.7 ± 0.5	3.6 ± 0.5	0.4
Gestational age (week)	39.9 ± 1.4	40.3 ± 1.1	0.3
Birth weight for gestational age (Z-score)	0.25 ± 0.99	−0.18 ± 1.04	0.2
Glomerular tuft volume (μm^3^ ×10^6^) ^c^	1.97 ± 0.77	2.37 ± 0.82	0.1
Glomerular tuft area (μm^2^)	12,522 ± 3410	14,036 ± 3502	0.1
Glomerular tuft diameter (μm) ^c^	125.0 ± 18.2	132.7 ± 16.8	0.2
Glomerular Density (N per 10^6^ μm^2^)	3.29 ± 1.40	2.96 ± 0.85	0.4

^a^ Glomerular tuft volume and glomerular tuft diameter was calculated based on the measured glomerular tuft area (as described in detail in the methods section)

### Glomerular area against birth weight-related and clinical characteristics

In order to further analyse the associations we conducted linear regression statistics to investigate associations between glomerular area and birth-weight and clinical variables. In these analyses we included both patients with IgAN and normal controls. Glomerular area was significantly higher in those with low birth weight in the adjusted model 2 analysis (a decrease of 1357 μm^2^ in glomerular area for every 1 kg increase in birthweight, *p*-value =0.01) but not in the primary model 1 analysis. Furthermore, maternal pre-eclampsia, gender and age at biopsy were significantly associated with glomerular area in both model 1 and 2 analyses, whereas body weight, BMI and eGFR at biopsy associated with glomerular area only in model 1 analysis (Table 4).

**Table 4:** Linear associations between glomerular area and birth related and clinical characteristics, Norway (1988–2013)

	Model 1 β-coefficient^a^	p-value	Model 2 β-coefficient^b^	*p*-value		
Birthweight (Kg)	− 1143	0.06	−1357	0.01		
Gestational age (Weeks)	− 141	0.3	−198	0.1		
Birth weight for gestational age (Z-score)	− 711	0.08	− 521	0.2		
Maternal preeclampsia (Yes/No)	4305	0.02	3577	0.046		
Gender (male vs female)	1736	0.05	1849	0.03		
Age at biopsy (10 years)^c^	1368	0.007	1418	0.04		
Body weight at biopsy (10 kg)^c^	668	0.004	491	0.1		
Body mass index at biopsy (kg/m^2^)	238	0.02	141	0.6		
eGFR (10 ml/min/1.73m^2^)^c^	− 530	0.01	− 297	0.1		
Systolic BP (10 mmHg)^c^	159	0.5	− 164	0.54		
Urinary Protein (g/d)	132	0.7	154	0.6		

^a^ Model 1 adjusted for IgA nephropathy diagnosis at biopsy

^b^Model 2 adjusted for IgA nephropathy diagnosis at biopsy, gender and age at biopsy

^c^ Body weight at time of biopsy, eGFR and systolic BP are given per 10 unit increase to give meaningful coefficients, otherwise one unit was used for other variables

**Fig. 1:**
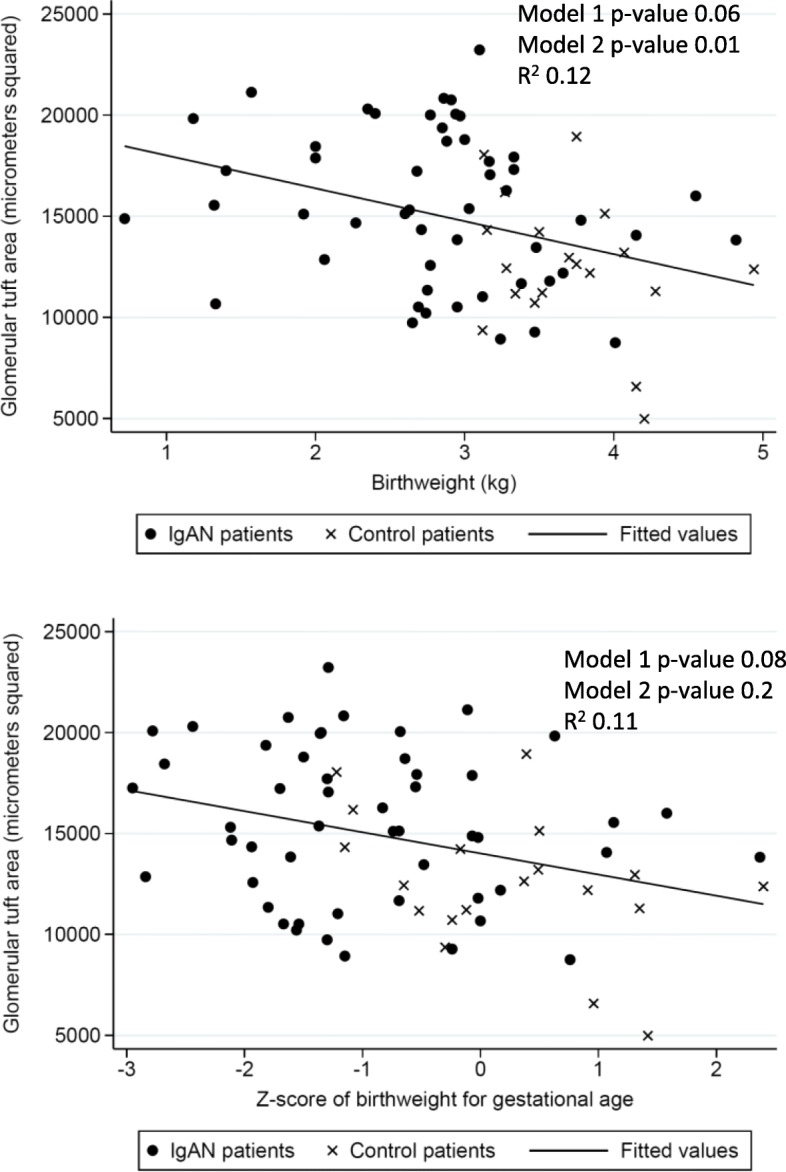
Relationship between Glomerular Area and birthweight and z-score of birthweight for gestational age. Model 1: Analysis adjusted for IgA nephropathy. Model 2: Analysis adjusted for IgA nephropathy, Age and Gender. *P*-values in figure given for the linear association between the two variables on x-axis and y-axis. Correlation coefficients R^2^ given for univariate correlation between the two variables on x-axis and y-axis

**Fig. 2:**
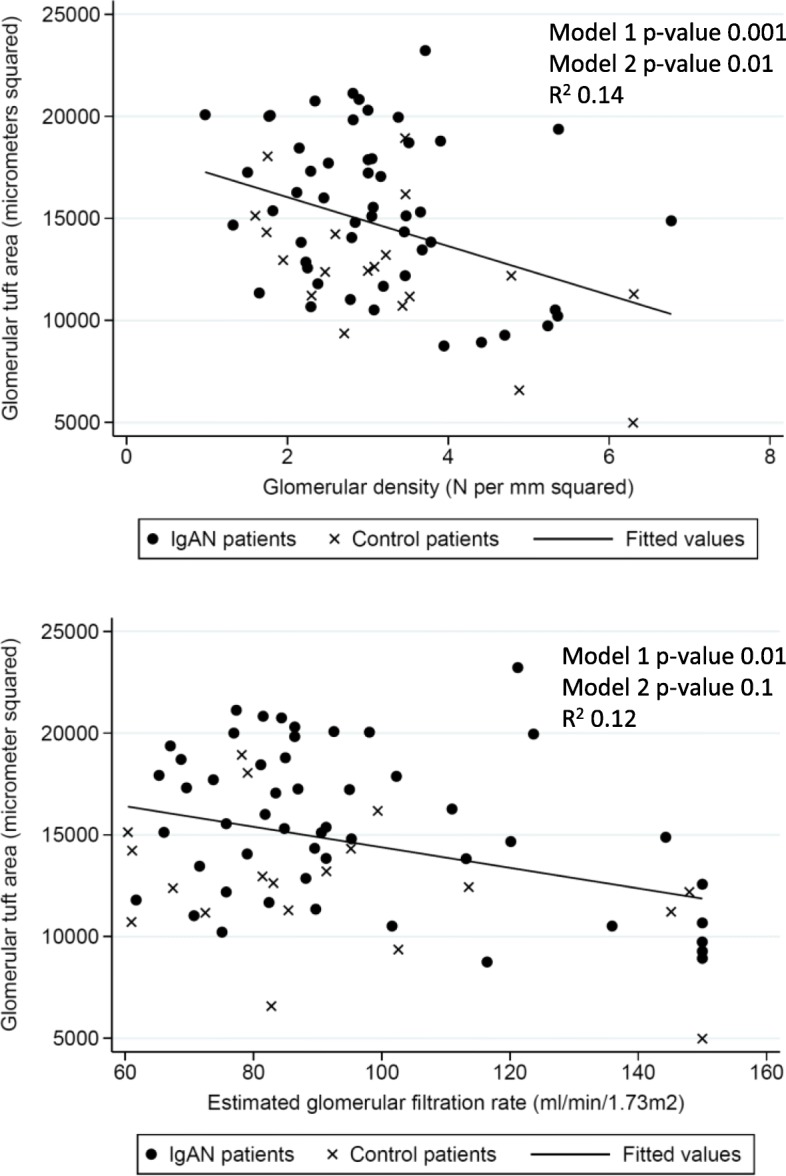
Relationship between Glomerular Area and Glomerular Density and Estimated Glomerular Filtration Rate. Model 1: Analysis adjusted for IgA nephropathy. Model 2: Analysis adjusted for IgA nephropathy, Age and Gender. P-values in figure given for the linear association between the two variables on x-axis and y-axis. Correlation coefficients R^2^ given for univariate correlation between the two variables on x-axis and y-axis

In Fig. 1 we show the inverse relationships between glomerular area and birthweight and z-score of birthweight for gestational age. In Fig. 2 we show the inverse relationships between glomerular area and glomerular density and eGFR.

## Discussion

The present study shows that young IgAN patients who were born with LBW and/or SGA had larger glomerular area at time of diagnosis than IgAN patients born with normal birth weight. The association was statistically significant in total cohort and in males. We consider larger glomerular area to be a marker of lower number of total glomeruli and the study thus suggests that larger glomeruli may at least in part, explain the higher risk of progressive renal disease in young IgAN patients with LBW and/or SGA in Norway [16]. Furthermore, considering that our cohort comprised young adults with preserved renal function (low-risk group), our findings may indeed underestimate the importance of this association. LBW and/or SGA may thus represent a basic vulnerability (first hit) among IgAN patients upon which other insults (immunology, hypertension etc. as second hits) may accelerate progressive nephropathy.

As described, LBW and/or SGA were associated with larger glomeruli at time of diagnosis of IgAN. Importantly, this association showed a dose-response correlation between birthweight and glomerular area. Larger glomeruli were also associated with lower eGFR. Previous studies have shown that glomerular size is a sensitive measure of total kidney glomerular number [18] and is proposed to be among the adaptive compensatory glomerular changes for congenital nephron deficit [17, 18, 33]. LBW has also been associated with low nephron number [34] and higher blood pressure [35] as well as microalbuminuria and low glomerular filtration rate in those born very premature and after intrauterine growth retardation [36]. In relation to IgAN in particular, a previous study has shown that progressive IgAN is associated with reduced glomerular density and increased glomerular volume [37]. The present study thus links these findings and suggests that LBW and/or SGA increase risk of progressive IgAN, mechanisms might involve lower glomerular number or higher blood pressure [1, 38]. These mechanisms might be important also in other kidney diseases as we previously have shown that LBW was associated with higher risk of ESRD in general, and also of ESRD due to glomerular disease [7, 39]. In the present study, LBW was defined by the 10th percentile in our previous study and corresponded to 2.93 kg for men and 2.69 kg for women [16]. As we observed a dose-response relationship between birth weight and glomerular size, we hypothesize that differences might have been larger if we had used a lower cut-off (WHO definition of LBW is < 2.5 kg). Neither LBW alone nor SGA alone were however significantly associated with larger glomeruli in Table 1 and we therefore decided to combine these groups in the remaining analyses. SGA and LBW may however partly be explained by different pathophysiological mechanisms and it is uncertain which is most strongly associated with later development of kidney disease [16]. As differences between groups with LBW and/or SGA in Table 1 were negligible we believe that this is the best approach. It is interesting to note that the group with SGA but not LBW (mean birth weight of 2.9 kg) had nearly the same glomerular area as the groups with LBW (mean birth weights of 1.9 and 2.4 kg). It should however be noted that in the comparison of only IgAN patients with vs without LBW (excluding the group with SGA and without LBW), glomerular area was statistically significantly different. Whether birthweight or birthweight for gestational age represents the most powerful predictor of later kidney disease and morphology must be analysed further in future studies.

Brenner postulated that congenital nephron deficit would lead to large glomeruli with glomerular hyperfiltration that would progressively increase risk of glomerular damage. A previous study has reported higher mean percentage of sclerotic glomeruli among IgAN patients who had suffered intra-uterine growth retardation [20]. Further, Ikezumi et al. reported association between LBW and development of focal segmental glomerulosclerosis in children and proposed that this was probably related to glomerular prematurity and podocytopenia [38]. In fact, podocytopenia has been associated with increased disease severity in IgAN [40] and in turn, compensatory podocyte hypertrophy has been associated with progressive glomerulosclerosis [41]. In our paper, there were non-significant trends towards more glomerular sclerosis in those with low birth weight but the degree of glomerular sclerosis was mild in our study as we selected patients with preserved kidney function. In our opinion, our study supports the Brenner hypothesis that large glomeruli with hyperfiltration is a link between low birth weight (as a marker of congenital nephron deficit) and progressive kidney disease.

As mentioned above, the association between LBW and larger glomeruli was significant in males but not females. In our previous paper [16] on IgAN, we reported that the association between LBW and ESRD was strong and significant in males but not females. The present study included a slightly higher proportion of female IgAN patients (53%) as compared to a previous study from the same registry (26%) [42]. These findings of a possible gender difference are interesting and warrant further investigation to supplement previous human and experimental studies that also have suggested that females might be protected against both progressive nephropathy [43–45] and intrauterine impaired nephron endowment or effects thereof [46].

In adjusted analysis, maternal preeclampsia was associated with a significantly larger glomerular area. Previous studies have shown that pre-eclampsia is associated with placental insufficiency and increases risk of LBW and SGA offspring [17, 47]. The present study suggests that the associated placental insufficiency might have especially important effects on kidney development. This finding is however limited by the small number of observations in this study (only four patients had a mother with preeclampsia) and should thus be interpreted with caution.

In this study, we report on glomerular morphology from kidney tissue specimens obtained from patients using percutaneous kidney biopsy needle as part of the routine clinical care. This method yields only a limited sample of glomeruli as compared to stereological methods using the physical dissector/fractionator method which is considered as the gold standard. Such stereological methods e.g. Cavalieri, Weibel-Gomez, Maximal Planar Area or Dissector Principle yield accurate estimation for glomerular size and density but are laborious, time consuming, costly and require the whole kidney tissue and are therefore of limited accessibility for routine studies [48, 49]. A previous study has illustrated that reliable mean estimates of glomerular size can be obtained by measuring 9 or more glomeruli; in white patients, measuring fewer than 6 glomeruli reduced the precision [32]. In the present study, even though some patients had fewer than 6 glomeruli, and fewer glomeruli would tend to lower precision of the mean, the glomerular size estimates were statistically significant between groups. Also, results for glomerular area were comparable in a sensitivity analysis where only patients with 9 glomeruli or more were included, although it did not reach statistical significance due to lower N. We would thus argue that estimates of glomerular area could be obtained also by measuring fewer glomeruli in studies with limited number of available patients and tissue.

## Conclusion

In the present study we have shown that among young IgAN patients with preserved renal function, impaired intrauterine growth was associated with larger glomerular area as a sign of congenital nephron deficit. This could in part explain the increased risk of progressive kidney disease in individuals born with LBW or SGA. Further larger studies should investigate why this effect seems to be more important in males than females and in population with higher frequency of offspring birthweight < 2.5 kg. However, the non-significant trend towards larger glomeruli may mean other plausible theories such as pre-diagnosis loss of glomeruli with compensatory hypertrophy may not be ruled out as additional/alternative explanations to what we observe in this study. There seems to be sufficient evidence to argue that consideration of birth weight is an important part of the clinical history of patients with chronic kidney disease.

### Strengths

The strength of this study is that we directly test whether the Brenner hypothesis could explain the increased risk of progressive IgAN that was seen in our previous study [16] and that we test this in IgAN patients with preserved kidney function at an early stage of the disease.

### Limitations

The study is however limited by the fact that percutaneous kidney biopsy method samples only procures a limited number of glomeruli from a small portion of the kidney that may not adequately represent the entire kidney tissue, especially when we consider the fact that glomerular size varies within different kidney regions [50].

## Additional file

Additional file 1: **Figure S1.** Flowchart for Selection of Cases and Controls. (DOCX 39 kb)

## Abbreviations

BMI: Body Mass Index
EGFR: Estimated Glomerular Filtration Rate
ESRD: End Stage Renal Disease
GA: Glomerular Tuft Area
GD: Glomerular Density
GV: Glomerular Volume
IDMS: Isotope Dilution Mass Spectrophotometer
IgAN: IgA Nephropathy
LBW: Low Birth Weight
MEST: Mesangial hypercellularity, Endocapillary proliferation, Segmental sclerosis, Tubular atrophy
SGA: Small for Gestational Age
SPSS: Statistical Package for Social Sciences
