# Supplementary material for: Low birth weight associates with glomerular area in young male IgA nephropathy patients
Source: BMC Nephrol. 2018 Oct 22;19:287. doi: 10.1186/s12882-018-1070-7 (PMC6198493; doi:10.1186/s12882-018-1070-7)
Supplement: Supplementary file 1 — Figure S1. Flowchart for Selection of Cases and Controls. (DOCX 39 kb) [file 12882_2018_1070_MOESM1_ESM.docx]

**Supplemental Fig 1: Flowchart for Selection of Cases and Controls**

**Eligible Controls**

-Registered BW in the MBR

- Normal findings on kidney biopsy (NKBR)

- eGFR>60ml/min/1.73m^2^

**Eligible Patients**

-Registered BW in the MBR

- Diagnosis of IgAN (NKBR)

-eGFR>60ml/min/1.73m^2^

- No development of ESRD

-

**IgAN Cases;**

**No LBW, No SGA**

(n=20 selected of
 230 eligible patients)

**IgAN Cases;**

**LBW but not SGA**

(n=13 of 13 eligible)

**IgAN Cases;**

**Not LBW but SGA**

(n=15 of 15 eligible)

**IgAN Cases;**

**Both LBW & SGA**

(n=14 of 14 eligible)

**Controls;**

**No LBW, No SGA**

Age-and-Sex matched to cases without LBW/SGA

(n=20)

Biopsy Tissue available for;

**Controls;**

**No LBW, No SGA**

(n=19)

**IgAN Cases;**

**No LBW, No SGA**

(n=12)

**IgAN Cases;**

**No LBW, No SGA**

(n=10)

**IgAN Cases;**

**No LBW, No SGA**

(n=11)

**IgAN Cases;**

**No LBW, No SGA**

(n=18)
